# Requirement of aggregation propensity of Alzheimer amyloid peptides for neuronal cell surface binding

**DOI:** 10.1186/1471-2202-8-29

**Published:** 2007-05-02

**Authors:** David A Bateman, JoAnne McLaurin, Avijit Chakrabartty

**Affiliations:** 1 Departments of Medical Biophysics and Biochemistry, University of Toronto, Toronto, ON, Canada; 2 Centre for Research in Neurodegenerative Diseases, Department of Laboratory Medicine and Pathobiology, University of Toronto, Toronto, ON, Canada

## Abstract

**Background:**

Aggregation of the amyloid peptides, Aβ40 and Aβ42, is known to be involved in the pathology of Alzheimer's disease (AD). Here we investigate the relationship between peptide aggregation and cell surface binding of three forms of Aβ (Aβ40, Aβ42, and an Aβ mutant).

**Results:**

Using confocal microscopy and flow cytometry with fluorescently labelled Aβ, we demonstrate a correlation between the aggregation propensity of the Alzheimer amyloid peptides and their neuronal cell surface association. We find that the highly aggregation prone Aβ42 associates with the surface of neuronal cells within one hour, while the less aggregation prone Aβ40 associates over 24 hours. We show that a double mutation in Aβ42 that reduces its aggregation propensity also reduces its association with the cell surface. Furthermore, we find that a cell line that is resistant to Aβ cytotoxicity, the non-neuronal human lymphoma cell line U937, does not bind either Aβ40 or Aβ42.

**Conclusion:**

Taken together, our findings reveal that amyloid peptide aggregation propensity is an essential determinant of neuronal cell surface association. We anticipate that our approach, involving Aβ imaging in live cells, will be highly useful for evaluating the efficacy of therapeutic drugs that prevent toxic Aβ association with neuronal cells.

## Background

Alzheimer's disease (AD) is a progressive neurological disorder that is the most prevalent form of age-dependent dementia [1]. The neuropathological features of AD include amyloid deposits, neurofibrillary tangles, and selective neuronal loss. The principle constituent of amyloid deposits is a peptide denoted amyloid β(Aβ), with the most abundant forms being 40 and 42 amino acid residues long and termed Aβ40 and Aβ42, respectively [2]. The endocytic pathway has been implicated in the secretion and production of Aβ [3,4]. Aβ is produced from sequential endoproteolytic cleavage of the amyloid precursor protein (APP). First, β-secretase cleavage occurs in the acidic late endosomes [5-7] and thereafter, γ-secretase cleavage liberates Aβ40/42 into the endosomal lumen [8,9]. The endosomal contents can be either secreted from the cell [10-12] or transferred to the lysosome [13]. Exposure of Aβ to endosomal pH has been found to induce various changes in its conformational and oligomeric states [14-16], with the formation of amyloid fibrils, and other oligomeric forms [17-21].

There is growing evidence that Aβ aggregation is the causal event in AD pathology. Amyloid deposits of Aβ found in the limbic and association cortices are surrounded by signs of neurodegeneration: dead or dying neurons, activated microglial cells, and reactive astrocytes [22,23]. In addition, Aβ-induced neurotoxicity has been demonstrated in numerous cell culture studies [24-26]. Moreover, transgenic mice expressing AD associated mutant human APP develop neuropathological lesions similar to those of AD patients. Immunization of these transgenic mice with Aβ42 aggregates reverses much of the neuropathology [27,28]. A proposed hypothesis explaining this phenomenon is that the immune system acts as a peripheral sink that traps Aβ and depletes it from the central nervous system [29]. These studies provide compelling evidence that extracellular Aβ is a significant contributor to neurotoxicity in AD.

The cell surface represents the first site of interaction between extracellular Aβ and neurons, and may be the location where the neurotoxic cascade is initiated. Studies on the neurotoxicity of Aβ indicate that aggregated Aβ is generally more toxic than monomeric Aβ [20,21,24,25,30]. Given that the state of aggregation affects the neurotoxic properties of Aβ, we have sought to determine whether the aggregation state also influences the interaction of Aβ with the surface of neuronal cells. We demonstrate that the surfaces of neuronal cells possess protein-rich sites that bind Aβ, and that aggregation competence is a critical requirement for cell surface binding.

## Results

### Aggregation propensity of Aβ is unaffected by TMR labelling

The studies reported here make use of tetramethylrhodamine (TMR) labelled Aβ (peptide sequences listed in Table 1), where the TMR group is located on the side chain of the N-terminal lysine. TMR was selected over other probes because it has been shown that TMR does not selectively partition into any particular subcellular organelle or microenvironment [31-33] and its fluorescence properties are ideal for confocal microscopy [32-34]. Furthermore, in a previous study, rhodamine (the parent compound of TMR) has been covalently attached to the N-terminus of Aβ40 for thermodynamic solubility measurements, and the solubility behaviour of this labelled peptide was similar to that of unlabelled Aβ40 [35]. We have also demonstrated that attaching a fluorescent label to the N-terminus of Aβ via a flexible glycine linker does not alter its amyloidogenic properties [19,36]. We note that the N-terminus of Aβ is more accessible and less involved in amyloidogenesis than other regions of the sequence [37]; thus, fluorescent labelling of the N-terminus is likely to have the least influence on amyloidogenesis.

**Figure 1:**
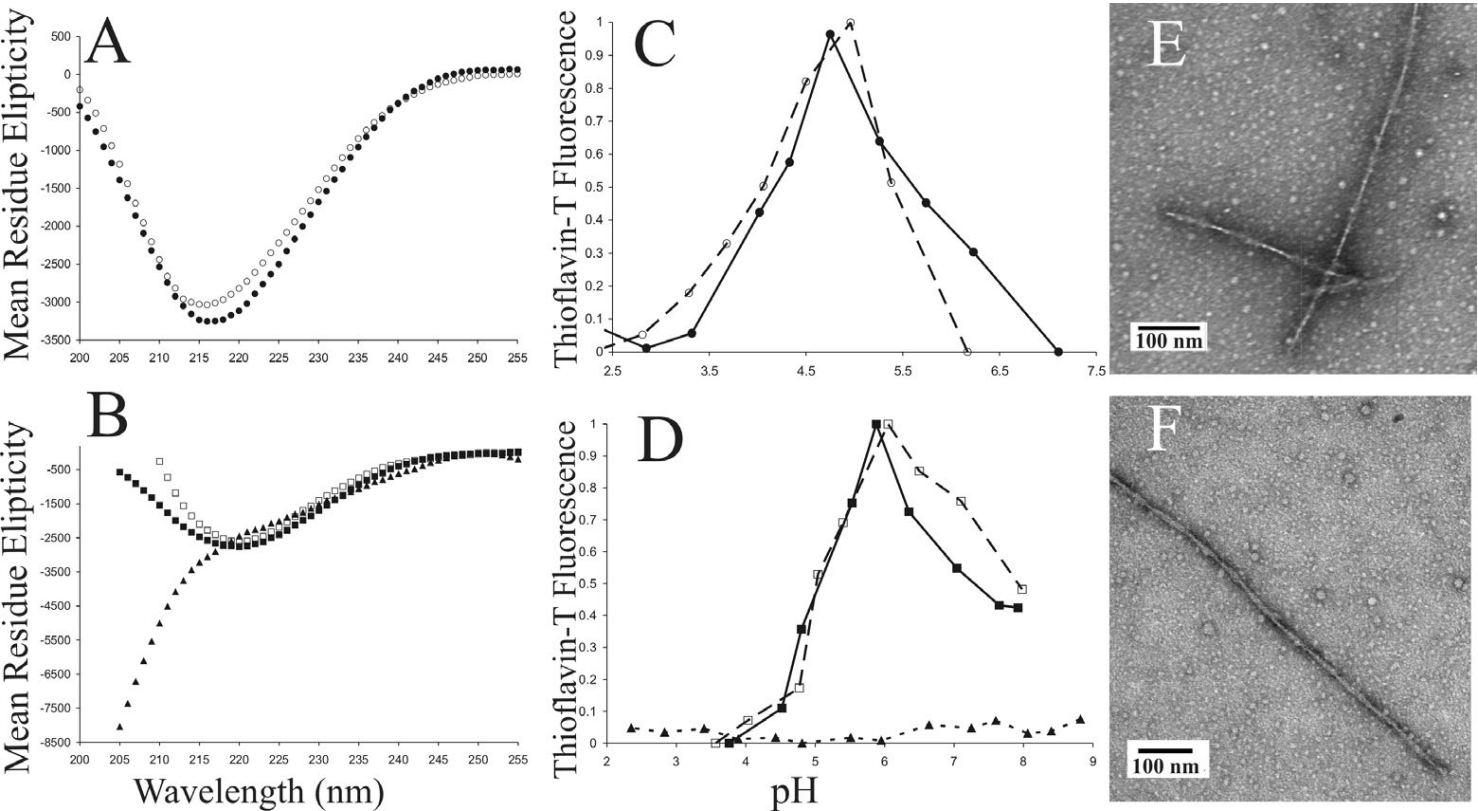
**Comparable aggregation properties of TMR-labelled and unlabelled Aβ**. (A) Similar β-sheet conformation of TMR-labelled Aβ40 (closed circles) and unlabelled Aβ40 (open circles) was observed using circular dichroism. (B) Similar β-sheet conformation of TMR-labelled Aβ42 (closed squares) and unlabelled Aβ42 (open squares) was found using circular dichroism. Unstructured conformation of TMR-labelled mutant peptide (closed triangles) is also shown. (C) Thioflavin-T fluorescence indicated similar pH-dependent aggregation profiles for both TMR-labelled Aβ40 (solid line, closed circles) and unlabelled Aβ40 (dashed line, open circles). (D) Thioflavin-T fluorescence also indicated similar aggregation profiles for both TMR-labelled Aβ42 (solid line, closed squares) and unlabelled Aβ42 (dashed line, open squares) as well as very little aggregation produced by the TMR-labelled mutant peptide (dotted line, closed triangles). Electron microscopy images of TMR-labelled Aβ40 and TMR-labelled Aβ42 amyloid fibrils are shown in (E) and (F), respectively, with 100 nm scale bars. The high similarity between labelled and unlabelled peptides suggests that the addition of the fluorescent label has no observable effect on the aggregation profile of Aβ.

We performed several biochemical characterizations of the TMR-labelled peptides used in this study, and we found that the labelled and unlabelled peptides behaved similarly. We found that labelled versions of both Aβ40 (Figure 1A) and Aβ42 (Figure 1B) exhibited identical circular dichroism spectra to their unlabelled counterparts, all possessing a negative band at 218 nm, which is characteristic of β-structure. We also studied a double mutant (F19S/L34P) of Aβ42 (hereon referred to as 'mutant peptide'), which was discovered through a GFP-based screen for mutations that reduce the aggregation propensity of Aβ42. These two particular mutations were shown to transform the highly aggregation-prone Aβ42 into a soluble unstructured monomeric peptide [38]. TMR-labelled mutant peptide was found to exhibit an unstructured conformation by circular dichroism (Figure 1B), even after storage for sixty days at room temperature (pH 6). We used thioflavin-T to assess the amyloidogenic potential of the peptides. Thioflavin-T is a dye known to shift its fluorescence from 430 nm to 490 nm upon binding specifically to the cross-β-structure of amyloids but not to the monomeric or small oligomeric complexes [39,40]. Using thioflavin-T fluorescence, we observed very similar pH-dependent fluorescent emissions for both labelled and unlabelled Aβ40 (Figure 1C) and Aβ42 (Figure 1D). Interestingly, the mutant peptide did not exhibit significant thioflavin-T fluorescence enhancement (Figure 1D). Negative stain electron microscopy revealed that TMR-labelled Aβ40 (Figure 1E) and TMR-labelled Aβ42 (Figure 1F) could produce characteristic unbranched amyloid fibrils. In sum, these data demonstrate that TMR-labelling does not alter the amyloidogenic properties of these peptides.

### Aggregation propensity is varied amongst Aβ isoforms

**Figure 2:**
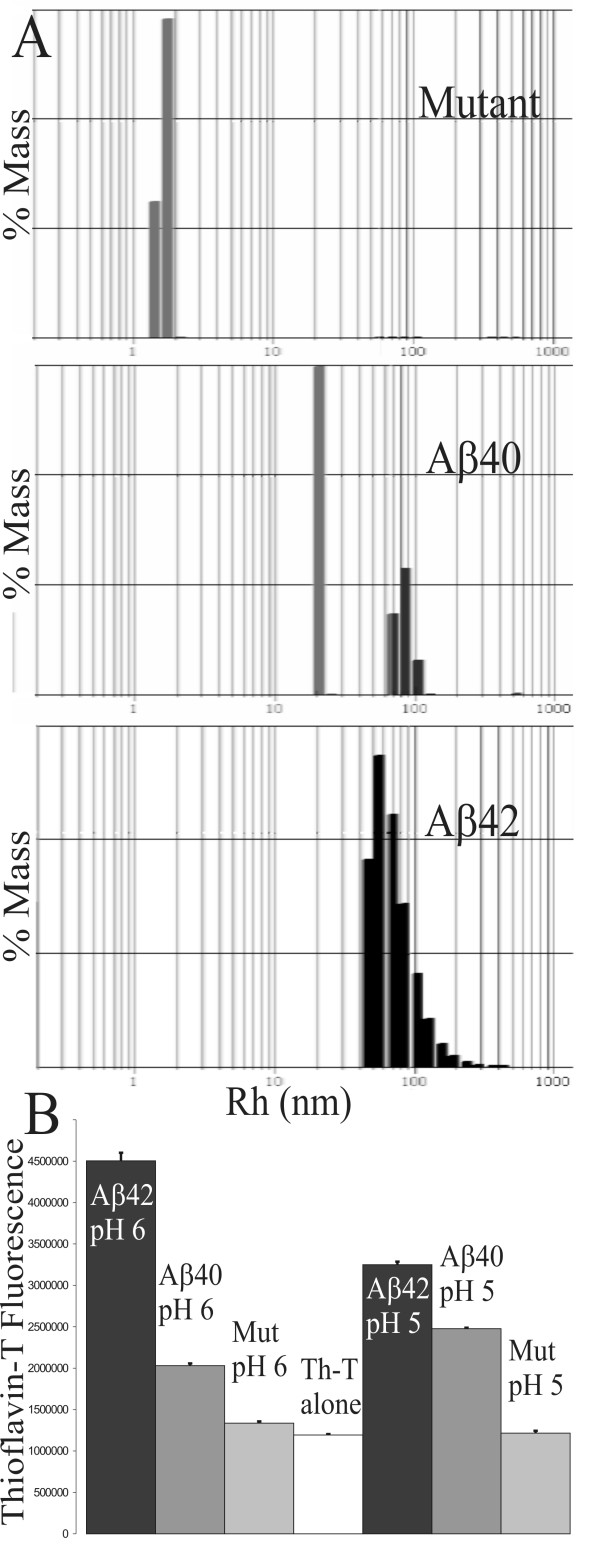
**Comparison of aggregation propensities of different TMR-labelled Aβ**. (A) Dynamic light scattering analysis indicated that very small particles (likely to be monomers) were present in the mutant peptide solution, two distributions of oligomers were present for Aβ40, and larger oligomers were present for Aβ42. (B) Aggregation marked by thioflavin-T fluorescence at pH 6 and pH 5 illustrated weak fluorescence for the mutant peptide (Mut), moderate aggregation for Aβ40, and the highest aggregation for Aβ42. The thioflavin-T alone sample represents the total emission peak area for a control sample not containing any Aβ. The data from these techniques indicates that the rank order of aggregation propensity is Aβ42 > Aβ40 > mutant.

The aggregation propensities of Aβ42, Aβ40, and the mutant peptide have been recently investigated [38,41,42]. We compared the TMR-labelled versions of these peptides and found that they follow the same order of aggregation propensity: Aβ42 > Aβ40 > mutant peptide. Dynamic light scattering was used to assess the degree of aggregation of the peptides after incubation for 90 minutes at room temperature, pH 6 (Figure 2A). The mutant peptide did not oligomerize under these conditions, Aβ40 formed a bimodal distribution of small and large oligomers with hydrodynamic radii of 10 and 85 nm, respectively, and Aβ42 formed large oligomers with radii centered around 55 nm but extending to particles with radii of several hundred nm. Thioflavin-T fluorescence was used to compare the extent of amyloidogenesis of these peptides at pH 6 and pH 5 (Figure 2B). These pH values (pH 6 and pH 5) were selected as they were found to induce the largest thioflavin-T fluorescence for Aβ42 (Figure 1D) and Aβ40 (Figure 1C). Under these conditions, the order of thioflavin-T fluorescence was: Aβ42 > Aβ40 > mutant peptide. As previously mentioned, we did not observe significant thioflavin-T fluorescence over a wide pH range for the mutant peptide (Figure 1D). The mutant peptide seemed aggregation incompetent under conditions that were conducive to forming amyloid fibres for Aβ42 and Aβ40. Our experimental results indicate that the rank order of aggregation propensity of the TMR-labelled peptides is the same as their unlabelled counterparts.

### Drastic differences in cell surface associations between Aβ isoforms

**Figure 3:**
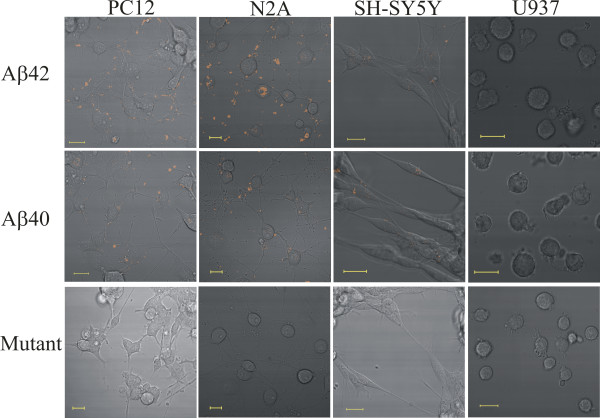
**Confocal microscopy images of various cell lines treated for 12 hours with TMR-labelled peptides**. All scale bars are 20 μm in length. The fluorescent emissions of TMR-labelled peptides are indicated in red. Aβ42 displayed the greatest cell surface binding among the peptides to the differentiated neuronal cell lines (PC12, N2A and SH-SY5Y). The mutant peptide was not observed to associate with any of the cell lines tested. None of the peptides tested associated with the U937 cells.

Using confocal microscopy to image TMR-labelled Aβ, we examined the association of the peptides to the surface of three model neuronal cell lines (PC12, N2A and SH-SY5Y) and one non-neuronal cell line (U937). Interestingly, we observed a correlation between aggregation propensity and peptide deposition on all three neuronal cell lines (Figure 3). After incubating the cells with peptide for 12 hours, Aβ42 was observed to associate strongly with all three differentiated neuronal cell lines in localized punctate regions. Aβ40 was found to exhibit a similar punctate association, but to a lesser extent than Aβ42. The mutant peptide, which is aggregation incompetent, did not associate to the surface of any of the cell lines tested. The apparent lack of cell surface association by the mutant peptide clearly supports the idea that cell surface association requires a specific peptide conformation or sequence and argues against non-specific interactions brought about by the TMR group [32-34].

The lack of Aβ association to the human lymphoma cell line, U937 (Figure 3) is noteworthy. Massiotti and Perlmutter [43] reported that U937 cells are resistant to Aβ induced cell death. We have also found them to be resistant to Aβ toxicity and incapable of binding Aβ over all exposure times tested (data not shown). To ensure that the lack of binding was not caused by degradation of Aβ by U937 cells, Aβ immunoblots were performed after exposure to U937 cells. The integrity of Aβ was maintained after exposure to U937 cells (see Additional file 1, Figure S2).

### Aggregation propensity of Aβ correlates with cell surface binding kinetics

**Figure 4:**
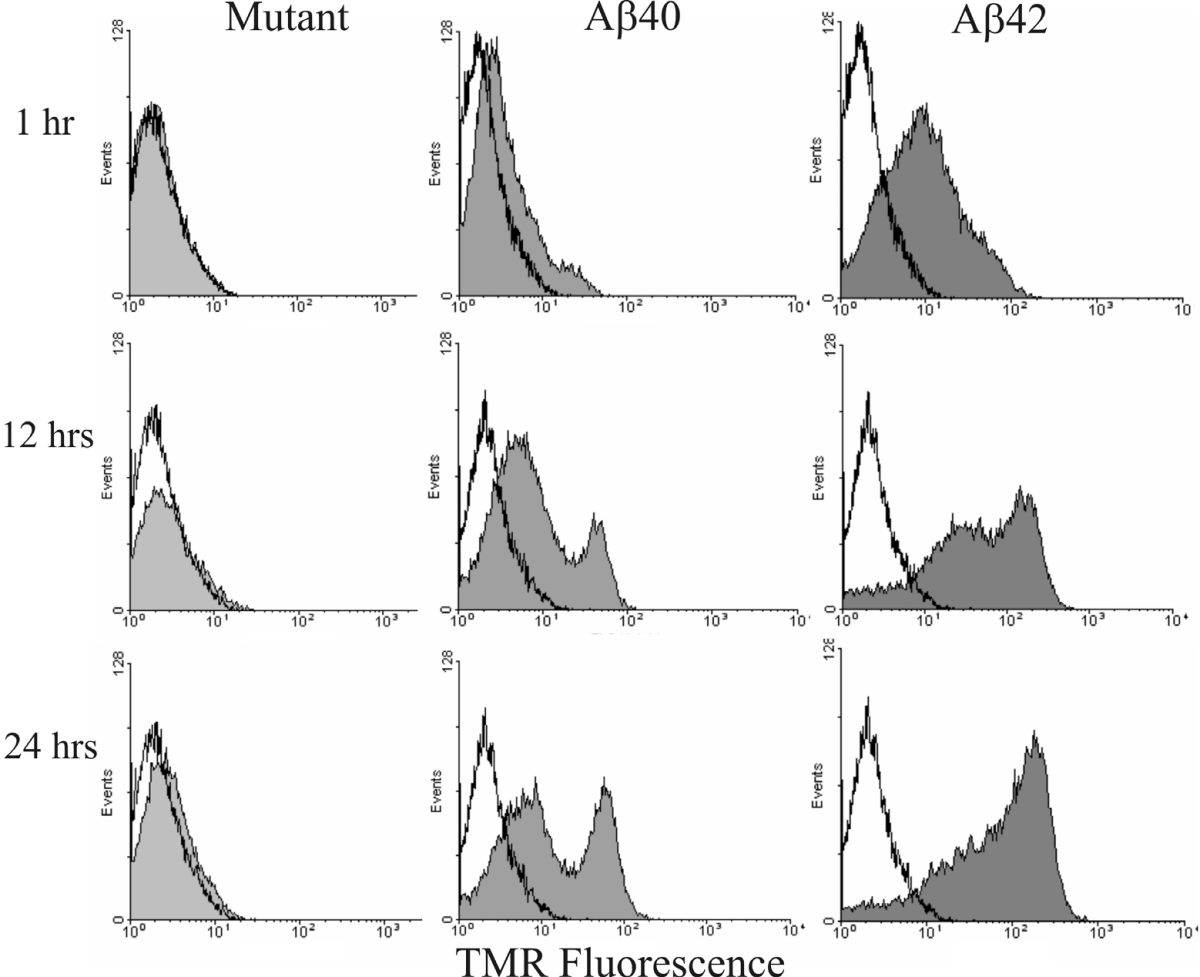
**Flow cytometry histogram plots showing association kinetics of TMR-labelled peptides with live NGF differentiated PC12 cells**. The thick black line represents untreated control cell autofluorescence. Aβ42 showed rapid cell association, whereas mutant peptide did not appear to significantly associate with the live cells. It is interesting to note the bimodal distribution that develops over time.

Having established an unambiguous link between peptide aggregation propensity and neuronal cell surface association, we investigated whether there is a similar correlation with cell surface binding kinetics. We treated NGF-differentiated PC12 cells with each peptide and monitored TMR fluorescence on live cells as a function of time by flow cytometry analysis. Dead cells were readily distinguished from live cells by staining with 7-AAD, a DNA binding dye that is excluded by live cells with intact membranes [44,45]. Figure 4 shows the striking differences in the levels of TMR florescence on live PC12 cells within only one hour of peptide exposure. Aβ42 clearly displayed the highest rate of cell surface association, followed by Aβ40. In contrast, the mutant peptide showed no apparent association with live cells.

**Figure 5:**
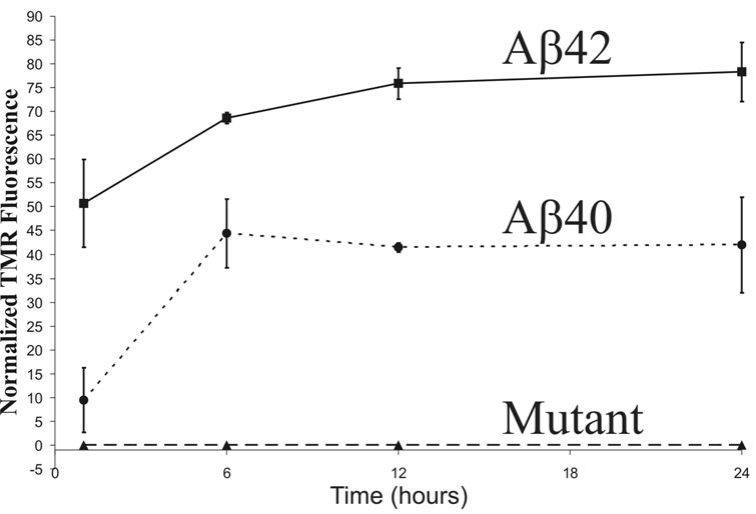
**Average kinetics of TMR-labelled peptide association with live NGF differentiated PC12 cells**. Aβ42 (solid line) revealed an initial rapid binding phase within the first hour, followed by a slower binding phase over the next 24 hours. Aβ40 (dashed line) did not display the initial rapid binding phase, but was otherwise similar to Aβ42. The mutant peptide (dotted line) did not interact with the live NGF differentiated PC12 cells. This finding suggests a correlation between aggregation propensity and cell association for Aβ.

Averaging multiple flow cytometry analyses and normalizing to the percentage of live cells labelled with TMR, we were able to closely study the differences in cell association kinetics displayed by each peptide (Figure 5). Aβ42 showed a fast binding phase (< 1 hour) in which the peptide bound ~50% of the live cells present. This was followed by a slower binding phase that occurred over a 24-hour period. The fast binding phase was completely absent in the binding kinetics of Aβ40. However, other kinetic features of Aβ40 binding were found to be similar to the slower binding phase of Aβ42. Notably, the mutant showed no measurable interaction with live NGF differentiated PC12 cells (Figure 5). The rate of Alzheimer amyloid peptides association with neuronal cells was directly related to their aggregation propensity, indicating that aggregation propensity is an essential determinant of both the rate and amount of cell surface association.

### Trypsin reduces cell surface binding of Aβ42

**Figure 6:**
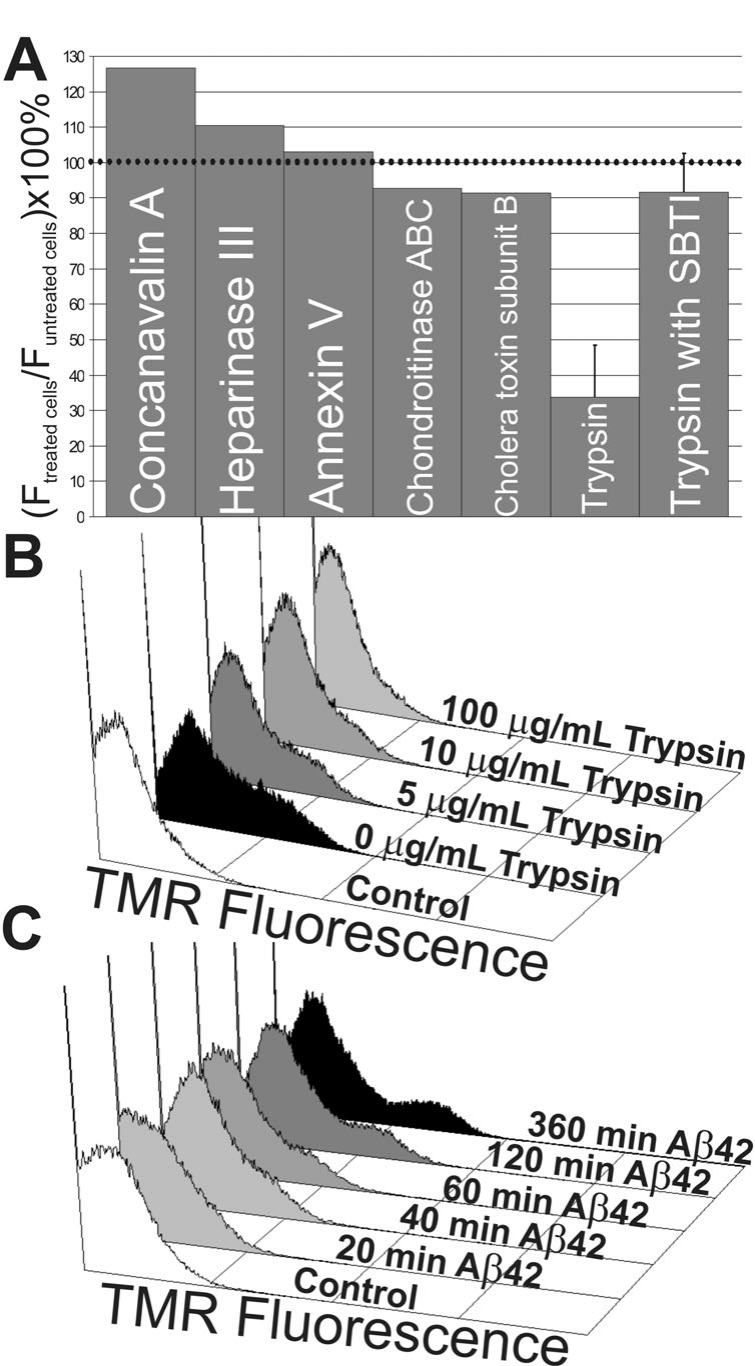
**Flow cytometry fluorescence results indicating trypsin significantly reduces Aβ cell surface binding**. (A) Cell surface treatments show that 10 μg/mL trypsin significantly decreased Aβ42 association to NGF differentiated PC12 cells, while treatments that target cell surface lipids or carbohydrates had no effect. SBTI-inactivated trypsin had little effect on Aβ42 association. (B) Concentration dependence of trypsin treated NGF differentiated PC12 cells with 0, 5, 10 and 100 μg/mL trypsin as indicated, followed by 1 hour Aβ42 treatment. (C) 10 μg/mL trypsin treatment of NGF differentiated PC12 cells demonstrate that Aβ42 association with the cell surface recovers over time.

To determine the molecular nature of the cell surface Aβ binding sites, we examined whether the following reagents blocked Aβ binding: concanavalin A, heparinase III, annexin V, chondroitinase ABC, cholera toxin subunit B, and trypsin (see Materials and Methods for details). Concanavalin A binds mannosyl and glucosyl residues [46,47], it can be used to block the potential binding of Aβ to these cell surface sugars. Since heparin sulphate and chondroitin sulfate have been suggested as possible binding sites for Aβ [48], the NGF differentiated PC12 cells were treated with heparinase and chondroitinase (in the presence of protease inhibitors) to remove these groups [49]. Fluorescein-labelled annexin V was used to block potential Aβ interactions with phospholipids. Annexin V preferentially binds to phosphatidylserine [50], however at high concentrations it non-specifically binds to any negatively charged phospholipids [51]. GM1 ganglioside has also been implicated as a potential Aβ binding molecule [52]. Fluorescein-labelled cholera toxin subunit B was used to block AβGM1 ganglioside interactions, as it is known to bind GM1 ganglioside and is used as a marker for lipid rafts [53-55]. All of the treatments mentioned above failed to block binding of Aβ to the surface of live NGF differentiated PC12 cells (Figure 6A), and co-staining of TMR and fluorescein signals was neither observed for annexin V nor cholera toxin subunit B in confocal and flow cytometry results (data not shown). The only cellular treatment that was observed to significantly decrease Aβ binding was trypsin digestion (Figure 6A), indicating that the binding sites are likely membrane proteins. We performed experiments to rule out the possibility that the reduction in Aβ42 binding is simply caused by degradation of Aβ42 by trypsin; after each trypsin treatment, trypsin was inactivated with excess of soybean trypsin inhibitor (SBTI) and the cells were washed thoroughly prior to adding TMR-labelled Aβ42. In addition, Aβ immunoblot analysis of the cell extracts and conditioned media were performed after trypsin treatments. No proteolytic degradation of Aβ was detected (see Additional file 1, Figure S3). Trypsin treatment in the presence of SBTI abrogated the effect of trypsin (Figure 6A), thus the blockage of Aβ binding is caused by the proteolytic activity of trypsin and not by some other process. Cellular trypsin treatments were also observed to be concentration dependent (Figure 6B), further supporting that trypsin treatment abolishes the cell surface binding of the peptide. Interestingly, Aβ binding was recoverable if the live trypsin-treated cells were allowed to sit for 2 – 6 hours in the absence of trypsin (Figure 6C). This result suggests that after trypsin removal, continued protein synthesis replenished the Aβ binding sites on the cell surface. Collectively, these findings provide strong evidence that neuronal cell surface proteins play a critical role in the Aβ binding process.

### TMR-labelled Aβ42 binds to unlabelled Aβ42 aggregates on the cell surface

**Figure 7:**
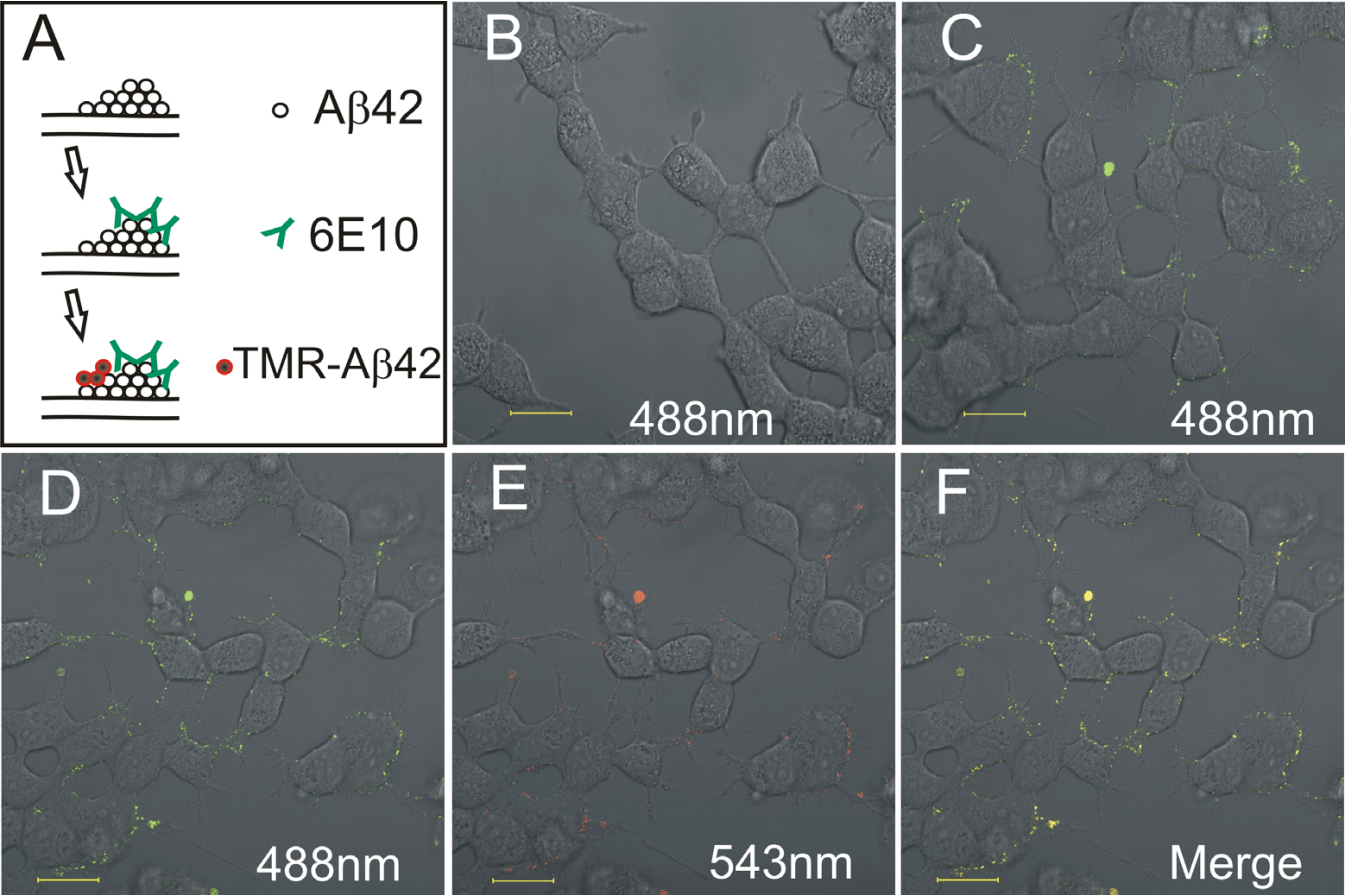
**Cell surface aggregation of Aβ**. All scale bars are 20 μm in length. (A) Schematic diagram indicating the order of cell surface treatments on NGF differentiated PC12 cells, separated by wash steps indicated by the arrows. (B) Confocal microscopy image of cells treated with 6E10 and secondary Alexa fluor 488 labelled antibody. The absence of staining in this control indicates the primary and secondary antibodies do not non-specifically bind to the cell surface. (C) Images of cells treated with 5 μM unlabelled Aβ42 followed by immunostaining with 6E10 and secondary Alexa fluor 488 labelled antibody. (D) Images of cells treated with 5 μM unlabelled Aβ42 followed by immunostaining, and treatment with 1.5 μM TMR-Aβ42 using 488 nm laser excitation. (E) Images of cells treated with 5 μM unlabelled Aβ42 followed by immunostaining, and treatment with 1.5 μM TMR-Aβ42 using 543 nm laser excitation. (F) Merge confocal microscopy image indicating regions of 6E10 antibody only in green, TMR-labelled Aβ42 in red and co-localized regions in yellow.

Our demonstration that aggregation propensity is a prerequisite property for cell surface binding suggests that cell surface proteins bind Aβ aggregates, and may serve as seeding sites that promote further Aβ aggregation on the cell surface. To test this possibility, we treated NGF-differentiated PC12 cells with unlabelled Aβ42 (see Methods). To visualize the unlabelled Aβ42 bound to the cell surface, the cells were immunostained with a monoclonal antibody (6E10) directed against the N-terminus of Aβ. Control experiments, in which 6E10 and secondary antibodies were applied to cells that were not treated with Aβ42, indicated that the antibodies do not bind nonspecifically to the cell surface (Figure 7B). However, immunostaining of cells treated with unlabelled Aβ42 (Figure 7C) showed a punctate staining pattern that was similar to that observed in cells treated with TMR-labelled Aβ42 (Figure 3). This similarity in staining patterns indicates that TMR-labelling of the peptide does not perturb the cell surface association. Sequential treatment of the cells with unlabelled Aβ42, immunostaining with 6E10 antibody, and TMR-labelled Aβ42 indicated co-localization of unlabelled Aβ42 immunostaining with TMR fluorescence (Figure 7D–F). This co-localization indicates that TMR-labelled Aβ42 binds to unlabelled Aβ42 aggregates on the cell surface. Thus, Aβ that is bound to the cell surface is capable of binding to other Aβ molecules from the extracellular environment.

## Discussion

Our investigation adapts a versatile imaging technique, and reveals a striking correlation between the aggregation propensity of Alzheimer amyloid peptides and their cell surface association kinetics with neuronal cells. Previous studies have shown that Aβ42 has a higher propensity to aggregate [41,42,56] and is the primary constituent in senile plaques [57,58]. However, the direct link between neuronal cell association and peptide aggregation propensity was unclear until now. Aβ40 is the primary product of proteolytic cleavage from the amyloid precursor protein [56]. We found that Aβ40 displays relatively slow association kinetics with neuronal cells (Figure 5) and would be more likely to be cleared *in vivo*, before initiating toxicity. On the other hand, Aβ42 associates rapidly with neuronal cells (Figure 5) and is thus more capable of initiating neurotoxicity and plaque formation. These observations offer insight into the early mechanism of AD onset seen with familial mutations that lead to an increase in Aβ42 [59]. Since Aβ42 associates with cells at such a rapid rate, this process may exceed the rate at which the aging macrophage machinery clears amyloid deposits, resulting in the senile plaque core found within the brain of Alzheimer patients. The link between aggregation propensity and cell association becomes more evident with the double mutant of Aβ42 [38]. This aggregation incompetent mutant peptide does not associate with the surface of any of the cell lines tested (Figure 3). This finding is evidence that cell surface association of Aβrequires a specific peptide sequence, conformation and/or aggregation state. Thus, Aβ aggregation propensity plays a critical role in initiation of the amyloid cascade.

Previous studies have used dyes, radioactive Aβ, and antibodies to study the interactions of Aβ with cell cultures [60-68]. Our results (Figure 3) reveal similar punctate staining as imaged recently with antibodies directed specifically against oligomeric structural forms of Aβ [65,66]. However, our technique allows images to be obtained with live cells throughout the progression of Aβ aggregation, and through its multitude of intermediate states. The major drawbacks of endpoint antibody detection with fixed cells or limited detection of specific conformations of Aβ are avoided by using our approach. This approach will be especially beneficial for developing therapeutic treatments that target specific conformations of Aβ, as the global effect of Aβ on the cell can be monitored.

The novel finding that U937 human lymphoma cells are resistant to cell surface binding by Aβ offers an excellent control for future therapeutic testing. This cell line was known to be resistant to Aβ toxicity [43], but the present findings provide the first biochemical evidence that U937 cells are missing the critical cell surface constituents that are responsible for interaction with Aβ and initiation of Aβ deposition on the surface of neuronal cells [69]. The lack of Aβ cell surface association was not caused by peptide degradation (see Additional file 1, Figure S2) over the course of the experiment but most likely resulted from the absence of particular cell surface proteins. Thus, this cell line provides a useful negative control for the identification of these proteins.

Our studies of Aβ imaging in live cells has uncovered the major role of membrane proteins as binding sites of Aβ40/42 on the neuronal cell surface (Figure 6A,C). These studies utilizing trypsin were carefully constructed to ensure that trypsin was completely inactivated with soybean trypsin inhibitor before peptide treatments were made. Immunoblot analysis confirmed that the trypsin-induced reduction in Aβ association was not caused by tryptic digestion of Aβ, as lower molecular weight Aβ derived peptides were not observed in the cell culture media or associated with the cell pellet fraction (see Additional file 1, Figure S3).

Many cellular components have been implicated to interact with Aβ [1,69,70], several of which are proteins [71-74]. Our results that Aβ binds preferentially to neuronal cells but not the non-neuronal cell line U937, indicates that the membrane protein binding sites are not present in this cell line. The membrane protein or proteins that bind Aβ have to be identified; however, there still remains the possibility that some of the membrane protein binding sites maybe unrelated to the amyloid cascade.

## Conclusion

Our experimental system of observing interactions of fluorescently labelled Aβ peptides with live neuronal cells provides a straightforward technique for evaluating potential therapeutic compounds for their ability to block interactions of Aβ with cell surface proteins and prevent the neurotoxic cascade elicited by Aβ. Our study has also revealed some of the molecular details controlling the interactions of these peptides with neuronal cells; namely, that aggregation propensity is a critical determinant of cell surface binding.

## Methods

All chemicals were purchased from Sigma-Aldrich (St. Louis, MO) unless otherwise stated. The following cell culture reagents were purchased from Gibco-Invitrogen (Burlington, ON): D-PBS, Dulbecco's modified Eagle's medium: nutrient mixture F-12 1:1 mixture (DMEM/F12), N2 supplement, nerve growth factor (NGF), penicillin and streptomycin.

### Peptide synthesis and purification

Aβ40 was prepared by solid-phase synthesis on a PerSeptive Biosystems 9050 Plus peptide synthesizer, as peptide-amides using Val-PEG-PS resin (PerSeptiveBiosystems). An active ester coupling procedure, employing O-(7-azabenzotriazol-1-yl)-1,1,3,3-tetramethyl-uronium hexafluorophosphate of 9-fluorenylmethoxycarbonyl amino acids, was used. Aβ42 and mutant Aβ42 were synthesized similarly using Ala-PEG-PS resin (PerSeptiveBiosystems). Before cleavage from the resin, the fluorophore, Nα-(9-Fluorenylmethoxycarbonyl)-Nε-tetramethylrhodamine-(5-carbonyl)-L-lysine (Molecular Probes, Eugene, OR) (abbreviated TMR) was coupled to the N-terminus via a glycine linker. The peptides were cleaved from the resin with a mixture of trifluoroacetic acid, thioanisole, m-cresol, and ethanedithiol (81:13:1:5 v/v). After incubation for twenty minutes at 25°C, the resin was removed by filtration and bromotrimethylsilane was added to a final concentration of 12.5 % (v/v). Following incubation for three hours, the peptides were precipitated and washed in cold ether. The crude peptides were dissolved in 6.5 M guanidine hydrochloride (pH 10) and purified by HPLC using a Superdex Tricorn 10/300 GL Peptide column (Amersham Biosciences, Piscataway, NJ) with 30 mM NH_4_OH running buffer. To maintain stock peptide solutions free from fibril seeds, solutions were stored at pH 10 and 4°C immediately after chromatographic separation of monomeric peptides. These conditions have been previously shown to maintain the monomeric state [19]. Peptide purity and identity was confirmed using both MALDI mass spectrometry and amino acid analysis. Concentrations of stock peptide solutions that were free of fibril seeds were determined using amino acid analysis and confirmed by either tyrosine absorbance (275 nm, ε = 1390 cm^-1 ^M^-1^) or TMR absorbance for labelled peptides (550 nm, ε = 92000 cm^-1 ^M^-1^).

### Circular dichroism

Aβ was added to 2 mM borate, 2 mM citrate and 2 mM phosphate buffer with adjusted pH. Spectra were obtained on an Aviv model 62DS circular dichroism spectrometer with a 0.1 cm path length quartz cuvette. Buffer spectra were subtracted from scans of each Aβ sample spectrum and the data were converted to mean residue ellipticity ([θ], degrees cm^2 ^dmol^-1^).

### Thioflavin-T assay

Peptide samples were prepared at 20 μM with 2 mM borate, 2 mM citrate and 2 mM phosphate adjusted to particular pH values and stored in the dark at 25°C for 20 hours. Fluorescence measurements were obtained after addition of five-fold molar excess of thioflavin-T and incubation at room temperature for thirty minutes. The excitation was at 440 nm and the emission peak was integrated from 475 nm to 495 nm.

### Election microscopy

Aβ solutions were prepared at 20 μM in 2 mM borate, 2 mM citrate and 2 mM phosphate, pH 6.5. Negatively stained fibrils were prepared by adding peptide solutions to charged pioloform, carbon-coated grids. After blotting and air-drying, the samples were stained with 1% phosphotungstic acid (pH 7). Electron microscopy images were acquired on a Hitachi H-7000 transmission electron microscope operated with a 75 kV accelerating voltage.

### Dynamic light scattering (DLS)

Hydrodynamic radius (Rh) measurements were made at 20°C with a DynaPro DLS instrument (Protein Solutions Inc., Piscataway, NJ). Peptides samples (pH 6) were incubated at room temperature for 90 minutes. Before measuring 90° light scattering intensity, samples were centrifuged at 12000 × g for three minutes. Particle translational diffusion coefficients were calculated from decay curves of autocorrelation of light scattering data and converted to hydrodynamic radius (Rh) with the Stokes-Einstein equation. Histogram of percentage mass versus Rh was calculated using Dynamics data analysis software (Protein Solutions Inc., Piscataway, NJ).

### Cell culture

Cell lines were maintained in DMEM/F12 containing 10% fetal bovine serum (HyClone, Logan, UT) with 100 units/mL penicillin and 100 μg/mL of streptomycin. To induce differentiation of PC12 cells, they were plated at 2.2 × 10^4 ^cells/cm^2 ^in Lab-tech chambered coverglass chambers and suspended in phenol red free DMEM/F12 containing N2 supplement and 10 ng/mL NGF. Cells were differentiated for 72 hours before media was replaced and peptide treatments were preformed. To induce differentiation of N2A cells, they were plated at 1.6 × 10^4 ^cells/cm^2 ^in Lab-tech chambered coverglass chambers and suspended in phenol red free DMEM/F12. Media was exchanged every 48 hours for six days before peptide treatments were performed. To induce differentiation of SH-SY5Y cells, they were plated at 1.6 × 10^4 ^cells/cm^2 ^in Lab-tech chambered coverglass chambers and suspended in phenol red free DMEM/F12 containing N2 supplement and 60 ng/mL NGF. Media was exchanged every 48 hours for six days before peptide treatments were performed. U937 cells were plated at 5.4 × 10^4 ^cells/cm^2 ^in phenol red free DMEM/F12 containing N2 supplement, 12 hours before peptide treatments were performed. All cells were maintained at 37°C in a humidified incubator with 5% carbon dioxide.

### Confocal microscopy

Fluorescent images were taken with a confocal laser-scanning system consisting of an LSM 510 Zeiss confocal microscope with a 40 × water immersion objective (n.a. 1.2) and HeNe laser with a 543 nm laser line. The temperature was regulated at 37°C using a heated stage and objective heater. The displayed images represent a single cross-section through the center of the cells. Cell viability and membrane integrity were confirmed with 20 μL/mL trypan blue exclusion.

### Flow cytometry

NGF differentiated PC12 cells treated with Aβ (1.5 μM) were washed once with cold D-PBS, followed by mechanical detachment with D-PBS. Collected cells were centrifuged (200 × g for 1 minute) and resuspended in cold flow buffer containing 5 mM EDTA and 1% BSA (Fisher Scientific, Nepean, ON) in D-PBS. Samples were immediately analyzed with a FACS Calibur flow cytometer (Becton Dickinson, Mississauga, ON). TMR-labelled samples were detected with 585/42 filter collecting 5 × 10^4 ^events per sample using 100–300 events/s flow rate. Cell viability was assessed with 25 μg/mL 7-Aminoactinomycin D (7-AAD) treatment for 5 minutes and detected with a long pass 650 nm filter. Data collection and analysis were performed using Cell Quest version 3.3 and figures were created using WinMDI version 2.8 software.

### Blocking the binding of Aβ to the surface of live PC12 cells

Various treatments to block or remove lipid, carbohydrate, or protein components from the surface of live PC12 cells were performed and their effects on Aβ binding was examined. NGF differentiated PC12 cells were treated with 100 ng/mL FITC labelled annexin V (to block negatively charged phospholipids) or 10 μg/mL FITC labelled cholera toxin subunit B (to block GM1 ganglioside) for 20 minutes before addition of Aβ42 (final concentration 1.5 μM) for two hours. NGF differentiated PC12 cells were treated with 250 μg/mL concanavalin A (to block mannosyl and glucosyl residues) for 20 minutes before addition of Aβ42 (final concentration 1.5 μM) for two hours. NGF differentiated PC12 cells were treated with 500 mU heparinase III (to remove heparin sulphate) or 500 mU chondroitinase ABC (to remove chondroitin sulfate) for 15 minutes in the presence of a proteinase inhibitor solution (10 mM EDTA, 5 mM Phenylmethylsulphonylfluoride, 50 mM sodium acetate in D-PBS) at 37°C [48,49]. The cells were then rinsed with media before treatment with Aβ42 (final concentration 1.5 μM) for an hour. To remove protein surface interactions, NGF differentiated PC12 cells were treated with 5 μg/mL, 10 μg/mL or 100 μg/mL trypsin (9590 units/mg) for 15 minutes, followed by addition of three molar equivalents of soybean trypsin inhibitor (SBTI 10000 units/mg) for 5 minutes, then media was exchanged and the cells treated with Aβ42 (final concentration 1.5 μM). NGF differentiated PC12 cells were also treated with a mixture of 10 μg/mL trypsin and 30 μg/mL SBTI for 15 minutes, followed by media exchange and one hour treatment with Aβ42 (final concentration 1.5 μM). After Aβ42 addition, treated cells and untreated control cells were analyzed using flow cytometry.

### Immunostaining Aβ on the surface of live PC12 cells

NGF differentiated PC12 cells treated with unlabelled Aβ42 (5 μM) for two hours at 37°C were washed once with cold blocking solution, containing 1% BSA (Fisher Scientific, Nepean, ON) and 0.1 % sodium azide in D-PBS, followed by 15 minute incubation at 4°C in cold blocking solution. Unlabelled Aβ42 was detected by incubation with a 12.5 μg/mL of 6E10 antibody (A1474, Sigma, St. Louis, MO), followed by incubation with 10 μg/mL of secondary rabbit anti-mouse Alexa fluor 488 conjugated antibody (A-11059, Gibco-Invitrogen, Burlington, ON) at 4°C in cold blocking solution. Following antibody removal and washing with cold blocking solution, NGF differentiated PC12 cells were treated with TMR-labelled Aβ42 (1.5 μM) in phenol red free DMEM/F12 containing N2 supplement and 10 ng/mL NGF for one hour at 4°C prior to confocal imaging.

## Abbreviations

7-AAD, 7-Aminoactinomycin D; AD, Alzheimer's disease; Aβ, amyloid β; APP, amyloid precursor protein; CD, circular dichroism; DMEM/F12, Dulbecco's modified eagle's medium: nutrient mixture F-12 1:1 mixture; D-PBS, Dulbecco's phosphate-buffered saline; EDTA, ethylenediaminetetraacetic acid; FITC, fluorescein isothiocyanate; GFP, green fluorescent protein; MALDI, matrix assisted laser desorption/ionization; NGF, nerve growth factor; N2A, neuroblastoma-2A; PC12, rat adrenal pheochromocytoma; SBTI, soybean trypsin inhibitor; TMR, tetramethylrhodamine.

## Authors' contributions

DAB designed the study, wrote the manuscript and performed all experiments. JM trained DAB in cell culture, provided the PC12 cell line and helped to draft the manuscript. AC conceived of the study and participated in its design and coordination. All authors read and approved the final manuscript.

## Supplementary Material

Additional file 1 Control experiments demonstrating covalent integrity of Aβ. The data provided are western blots indicating that degradation of Aβ was not observed under the conditions tested Click here for file

## Figures and Tables

**Table 1:** Sequences of Alzheimer amyloid peptides.

Name	Sequence
Aβ40	DAEFR HDSGY EVHHQ KLVFF AEDVG SNKGA IIGLM VGGVV
Aβ42	DAEFR HDSGY EVHHQ KLVFF AEDVG SNKGA IIGLM VGGVV IA
mutant Aβ42	DAEFR HDSGY EVHHQ KLV**S**F AEDVG SNKGA IIG**P**M VGGVV IA
TMR-Aβ40	(TMR)-KG DAEFR HDSGY EVHHQ KLVFF AEDVG SNKGA IIGLM VGGVV
TMR-Aβ42	(TMR)-KG DAEFR HDSGY EVHHQ KLVFF AEDVG SNKGA IIGLM VGGVV IA
TMR-mutant Aβ42	(TMR)-KG DAEFR HDSGY EVHHQ KLV**S**F AEDVG SNKGA IIG**P**M VGGVV IA
