# Supplementary material for: Requirement of aggregation propensity of Alzheimer amyloid peptides for neuronal cell surface binding
Source: BMC Neurosci. 2007 May 2;8:29. doi: 10.1186/1471-2202-8-29 (PMC1871596; doi:10.1186/1471-2202-8-29)
Supplement: Additional file 1 — Control experiments demonstrating covalent integrity of Aβ. The data provided are western blots indicating that degradation of Aβ was not observed under the conditions tested [file 1471-2202-8-29-S1.doc]

Additional file 1: Control experiments demonstrating covalent integrity of Aβ.

Supplemental Procedures:

*Immunoblotting* – Cell cultures were mechanically removed from Labtech chambered coverglass chambers and centrifuged (377 x g for 5 minutes). The supernatant (Med fraction) was removed and the cell pellets (Pel fraction) were suspended in equal parts of 9M urea and 2x tris-glycine loading buffer before boiling for 45 minutes. Samples were electrophoresed on 4-20% tris-glycine polyacrylamide gels (Invitrogen, Burlington, ON) and transferred by electroblotting to polyvinylidene fluoride (PVDF) membranes (PALL, Ann Arbor, MI). Tetramethylrhodamine was detected by incubation with a 1:1000 dilution of anti-tetramethylrhodamine antibody (A-6397, Invitrogen, Burlington, ON) in a solution of 5% (w/v) powdered milk, 0.05% (v/v) Tween 20 in PBS, followed by incubation with 1:5000 dilution of secondary goat anti-rabbit peroxidase conjugated antibody (401353, Calbiochem, La Jolla, CA). Unlabeled Aβ42 was detected by incubation with a 1:500 dilution of 6E10 antibody (A1474, Sigma, St. Louis, MO), followed by incubation with 1:5000 dilution of secondary goat anti-mouse peroxidase conjugated antibody (A4416, Sigma, St. Louis, MO). Visualization of peroxidase was performed with enhanced chemiluminescence reagent plus (RPN2132, Amersham, Buckinghamshire, UK).


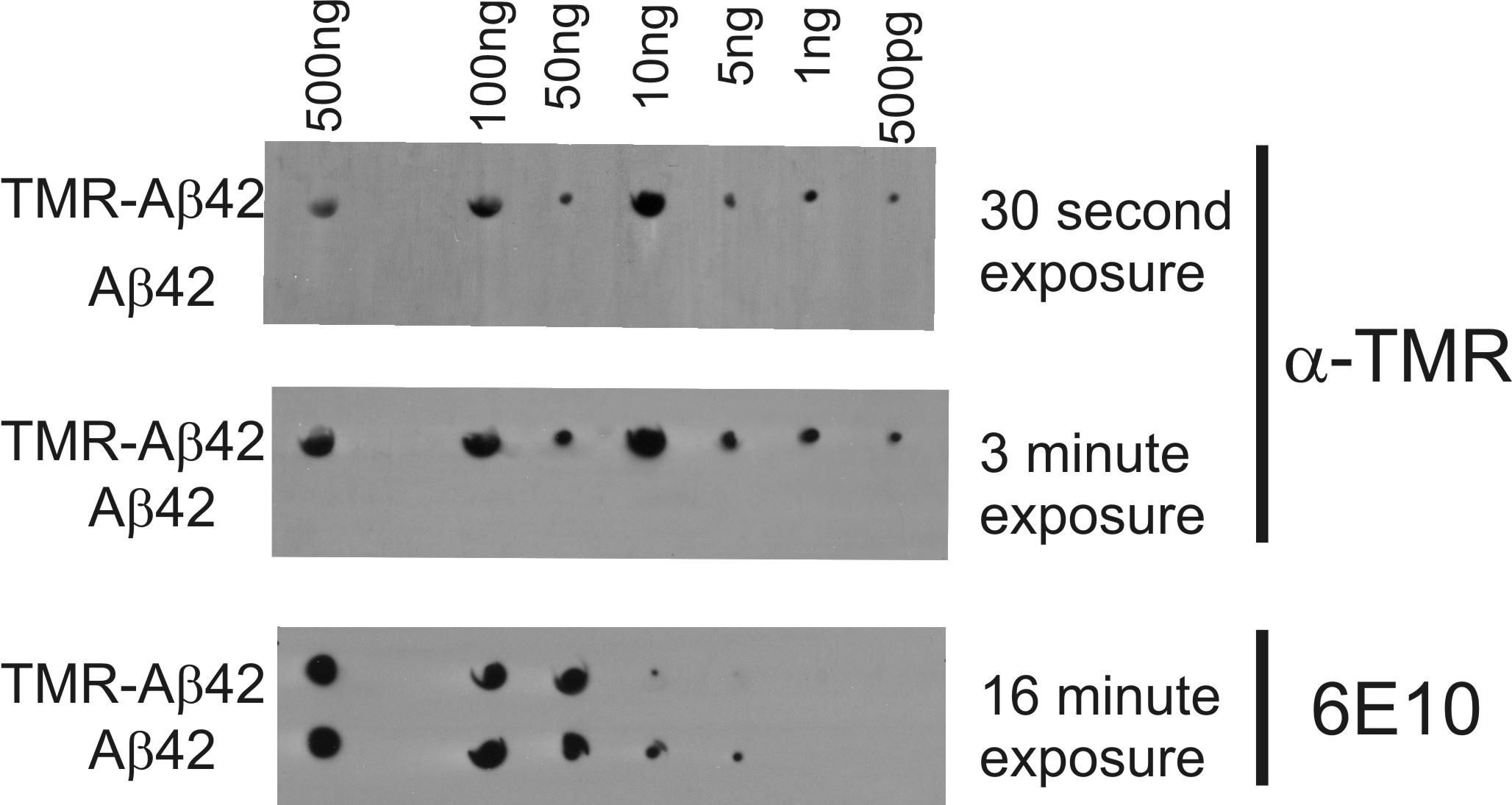


**Figure. S1. The anti-tetramethylrhodamine (α-TMR) antibody is highly sensitive to detecting tetramethylrhodamine.**  Labeled and unlabeled Aβ42 samples were spotted onto PVDF membrane strips and probed as indicated in supplemental procedures.

Additional file 1: Control experiments demonstrating covalent integrity of Aβ.


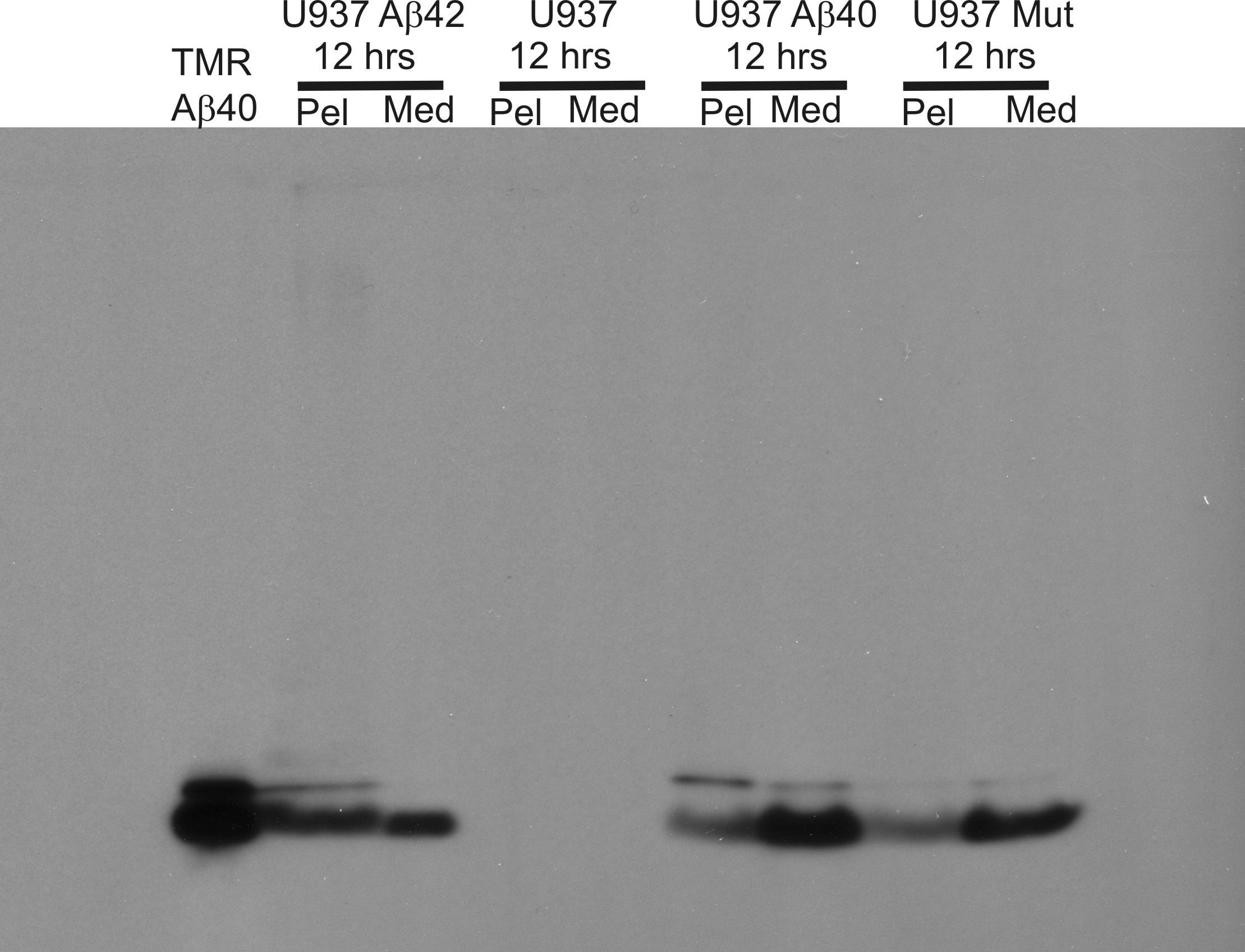


**Figure. S2.** **Absence of lower molecular weight Aβ bands indicates peptide integrity is maintained with U937 cells.** The highly sensitive anti-tetramethylrhodamine antibody indicates the presence of full-length Aβ in the media (Med) after 12 hour treatment with U937 cell line. The weak signal indicated in cell pellet fraction (Pel) is mainly due to nonspecific uptake of Aβ by dead or dying cells. The faint upper band present in the lanes may represent SDS-stable dimers.

Additional file 1: Control experiments demonstrating covalent integrity of Aβ.


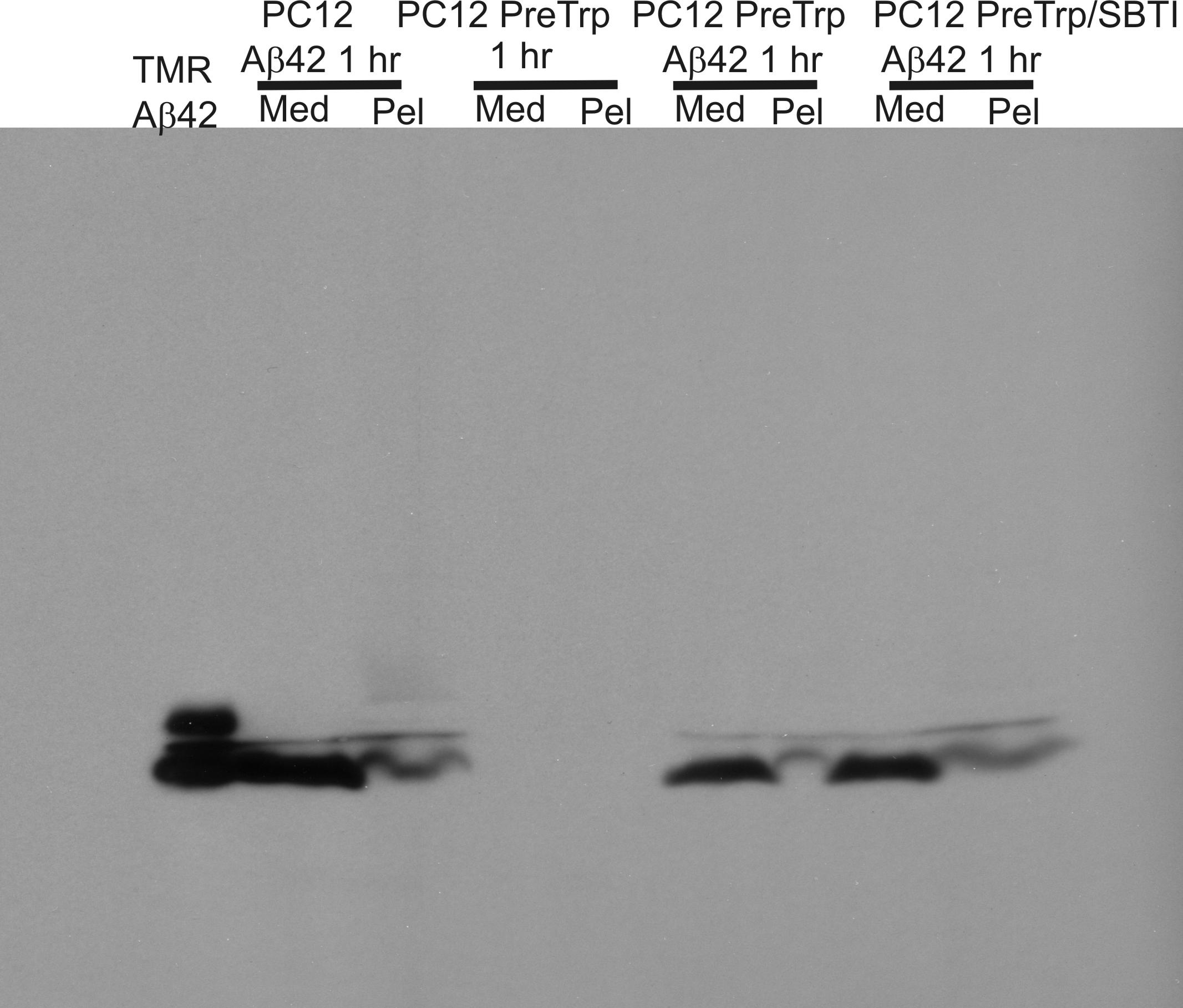


**Figure. S3.** **Absence of lower molecular weight Aβ bands indicates peptide integrity is not compromised with trypsin treatment.** The highly sensitive anti-tetramethylrhodamine antibody indicates the presence of intact peptides in the media (Med) and cell pellets (Pel).
